# B‑lines on lung ultrasound: a non‑invasive marker for short‑term outcomes after coronary artery bypass grafting

**DOI:** 10.1186/s13019-026-04573-0

**Published:** 2026-08-01

**Authors:** Nima Mohamadi Afrakoti, Mahan Khani, Sina Hemmati, Ali Ebrahiminasab, Reza Atef Yekta, Samaneh Hajy Molla Rabi, Elham Pagard, Mojtaba Marashi

**Affiliations:** 1 Department of Anesthesilogy, Dr Ali Shariati Hospital, Tehran University of Medical Sciences, Tehran, Iran; 2 Tehran University of Medical Sciences, Tehran, Iran; 3 Department of Neurology, Dr Rajaee Hospital, Alborz University of Medical Sciences, Alborz, Iran; 4 Shariati Hospital, Cardiovascular Surgical ICU, Tehran University of Medical Sciences, Tehran, Iran

**Keywords:** Coronary artery bypass grafting, Lung ultrasound, B-lines, Pulmonary congestion, Postoperative complications, Critical care monitoring, Cardiac anesthesia, Mortality, Prognosis

## Abstract

**Background:**

Postoperative pulmonary complications increase morbidity and mortality after coronary artery bypass grafting (CABG). Lung ultrasound detection of B-lines is an established method to quantify pulmonary congestion, and while B-lines have been studied as predictive markers in heart failure and general critical care settings, their role in predicting outcomes specifically for post-CABG patients remains inadequately defined. This study aimed to evaluate the association between B-lines detected on postoperative lung ultrasound and 30-day mortality and hospital length of stay in patients undergoing isolated CABG.

**Methods:**

This prospective cohort study was conducted in a single-center intensive care unit between 2023 and 2024. A total of 148 adult patients scheduled for elective CABG were enrolled. Lung ultrasound was performed on postoperative days 1 (POD1) and 3 (POD3) using a standardized 8-zone protocol to quantify B-lines. The primary outcomes were all-cause 30-day mortality, assessed via hospital records and telephone follow-up, and total hospital length of stay. Univariate and multivariate logistic and linear regression analyses were used to identify predictors.

**Results:**

The mean patient age was 60.15 ± 9.8 years, and 57% were male. Within 30 days, 22 patients (15%) died. In the multivariate logistic regression, the number of B-lines on POD1 was an independent predictor of 30-day mortality (Adjusted Odds Ratio: 1.090, 95% CI: 1.018–1.167, p = 0.013), indicating a 9% increase in the odds of death for each additional B-line. In contrast, the B-line count on POD3 was not significantly associated with mortality. B-line counts on both days were also not associated with hospital length of stay. Instead, higher body mass index (β = 0.22, p = 0.022) and a history of smoking (β = 2.20, p = 0.020) were independently associated with longer hospitalization.

**Conclusions:**

Early postoperative pulmonary congestion, as quantified by B-lines on POD1 lung ultrasound, is an independent predictor of 30-day mortality after CABG. Routine bedside lung ultrasound on the first day after surgery can serve as a non-invasive tool to stratify high-risk patients, potentially allowing for earlier intervention to optimize fluid status and improve outcomes.

## Background

Postoperative pulmonary complications are a major contributor to morbidity, mortality, and healthcare burden among surgical patients, particularly those undergoing cardiothoracic procedures. Early identification of pulmonary congestion in the postoperative period is crucial for optimizing fluid management and preventing adverse outcomes. Lung ultrasound (LUS) has gained increasing attention as a non-invasive, bedside imaging modality capable of detecting pulmonary edema through the identification of B-lines—vertical, hyperechoic artifacts originating from the pleural line that move with respiration and extend to the bottom of the ultrasound screen without fading [1].

B-lines are well-established markers of interstitial edema and extravascular lung water. In mechanically ventilated ICU patients, Anile et al. demonstrated a significant correlation between the number of B-line–positive lung quadrants and extravascular lung water measured by transpulmonary thermodilution (ρ = 0.61; p < 0.01) [2]. Sakamoto et al. further reported that B-line count correlates strongly with left ventricular filling pressures (r = 0.69; p < 0.001), and that incorporating B-lines into the ASE/EACVI diagnostic algorithm improved specificity for detecting elevated filling pressures [3]. Similarly, Imanishi et al. found that residual B-lines (≥ 6) at discharge predicted adverse outcomes in heart failure patients, including an increased risk of hospitalization [4].

The utility of B-lines extends into the postoperative setting as well. Rezende et al. recently demonstrated that elevated B-line scores within 24 h of pediatric cardiac surgery were significantly associated with extubation failure and prolonged mechanical ventilation, suggesting that early lung ultrasound assessment can effectively stratify respiratory risk in congenital heart disease patients. Their findings support the role of LUS in detecting postoperative pulmonary edema and guiding clinical decision-making in the immediate recovery phase [5].

In adult cardiac surgery populations, lung ultrasound has also shown potential utility. Charoenitthikun et al. investigated the relationship between B-line scores and pulmonary artery pressures in 32 patients who had undergone cardiac surgery, including 28 who received coronary artery bypass grafting (CABG). Their findings revealed statistically significant, though modest, correlations between B-line scores and mean pulmonary artery pressure (R^2^ = 0.36; p < 0.001), systolic PAP (R^2^ = 0.20; p = 0.01), and diastolic PAP (R^2^ = 0.10; p = 0.03). Moreover, this study did not assess associations between B-lines and clinical outcomes such as mortality or length of hospital stay [6].

Therefore, while the application of B-lines has been extensively studied in heart failure and critical care settings, their role as predictive biomarkers for early postoperative outcomes—particularly in CABG patients—remains inadequately defined. Existing studies are limited in scope, sample size, or are not specifically focused on CABG populations. Given the high incidence of fluid overload, transfusion requirements, and pulmonary complications following CABG, early, non-invasive markers of pulmonary congestion are of considerable clinical importance.

Our study aims to address this gap by prospectively evaluating the prognostic value of B-lines detected via postoperative lung ultrasound in predicting 30-day all-cause mortality and hospital length of stay among patients undergoing isolated CABG. By focusing on this specific surgical cohort, we seek to determine whether lung ultrasound can provide actionable insights for early risk stratification and improved postoperative care.

## Methods

### Study design and setting

This prospective cohort study was conducted in the Open Heart Intensive Care Unit (ICU) of Shariati Hospital, Tehran, Iran, between 2023 and 2024. All procedures were performed off-pump (without cardiopulmonary bypass). The study aimed to evaluate the prognostic value of B-lines detected via lung ultrasound in predicting short-term outcomes, specifically 30-day mortality and hospital length of stay, among patients undergoing coronary artery bypass grafting (CABG).

### Study population

Patients aged 18–80 years scheduled for elective CABG surgery were consecutively screened for eligibility. Exclusion criteria included a history of structural pulmonary disease (e.g., emphysema, pulmonary fibrosis, or primary/metastatic lung malignancies), pregnancy, prior cardiac surgery requiring redo sternotomy and perioperative myocardial infarction. A total of 157 eligible patients were enrolled following informed consent. Of these, 9 patients (5.7%) were lost to follow-up despite repeated contact attempts and were excluded from the dataset prior to any analysis, resulting in a final analytic sample of 148 patients. No imputation was applied to lost-to-follow-up cases.

### Data collection

Demographic information (age, sex, body mass index [BMI], smoking history), past medical history (e.g., hypertension, diabetes, chronic kidney disease), and baseline laboratory data (serum creatinine) were collected from medical records and direct interviews. Preoperative left ventricular ejection fraction (EF) was obtained from routine transthoracic echocardiography conducted by cardiology specialists.

### Lung ultrasound protocol

Postoperative lung ultrasound was performed on postoperative days 1 (POD1) and 3 (POD3) by a single board-certified anesthesiologist with ICU fellowship training, using a standardized 8-zone protocol. The 8 zones comprised the anterior and lateral chest wall bilaterally, each divided into upper and lower regions. B-lines were counted in each zone and summed to generate a total B-line score. A phased array (cardiac) probe was used for all examinations. All examinations were performed by the same operator throughout the study period, ensuring consistency and rendering inter-observer variability not applicable to this study design.

### Telephone follow-up protocol

Thirty-day postoperative mortality was assessed through a structured telephone follow-up protocol. Research staff collected up to three contact phone numbers from each patient or their close relatives before discharge. Follow-up calls were placed up to seven times per patient at various times: four attempts on non-holiday weekdays (10:00–18:00), followed by three additional attempts including weekends and holidays (09:00–19:00). If contact could not be established after all seven attempts, the patient was classified as lost to follow-up. A total of 9 patients (5.7%) were lost to follow-up.

### Outcome measures

Primary outcomes were all-cause mortality within 30 days postoperatively and the total length of hospital stay (from surgery until discharge). Mortality status was determined via hospital records or verified through telephone follow-up. Mortality was defined as all-cause death.

### Statistical analysis

Continuous variables were expressed as mean ± standard deviation (SD), and categorical variables as frequencies and percentages. Univariate logistic regression was used to explore associations with 30-day mortality, while univariate linear regression assessed predictors of hospital length of stay.

For multivariable models, we selected predictors based on both clinical relevance and statistical criteria. For 1-month mortality, POD1 B-lines, age, and sex demonstrated relatively low p-values in univariate analysis. Therefore, POD1 B-lines (primary variable of interest), preoperative EF (established prognostic factor), age (universal risk factor), and sex (based on known sex-based differences in CABG outcomes) were included in the multivariate logistic regression model.

Covariate selection was guided by both clinical relevance and univariate screening. All available clinical variables — including creatinine, diabetes, COPD, hypertension, and prior heart failure — were tested in univariate analysis.

For length of hospital stay, BMI and smoking history were selected due to their significance in univariate analysis and supporting literature. Preoperative EF, although not statistically significant, had a relatively low p-value suggestive of potential clinical relevance. POD1 B-lines were also included as the primary variable of interest.

Results of regression models are presented as odds ratios (OR) or beta coefficients (β) with corresponding 95% confidence intervals (CI). Statistical significance was defined as p < 0.05. All analyses were performed using SPSS version 27 (IBM Corp., Armonk, NY).

### Ethical considerations

The study protocol was approved by the Ethics Committee of Tehran University of Medical Sciences (Approval ID: IR.TUMS.SHARIATI.REC.1402.148). Written informed consent was obtained from all patients or their legal representatives. All lung ultrasounds and laboratory tests were provided free of charge. Patient confidentiality was maintained, and all data was anonymized for analysis and reporting.

## Results

### Demographic and clinical characteristics

A total of 148 patients were included in this study. The mean age was 60.15 ± 9.8 years, indicating a predominantly middle-aged to elderly population. The mean body mass index (BMI) was 24.72 ± 5.1 kg/m^2^, falling within the normal to overweight range.

Lung ultrasound on POD1 showed a mean of 19.01 ± 7.9 B-lines, which decreased to 11.76 ± 7.7 by POD3, suggesting a marked reduction following initial treatment. The mean preoperative left ventricular ejection fraction (EF) was 44.7% ± 8.8, reflecting moderate left ventricular systolic function. 30 patients (20.3%) had severely reduced EF (≤ 35%).

Comorbid conditions were prevalent across the cohort. Hypertension was the most common (84%), followed by diabetes mellitus (51%), a history of smoking (35%), chronic obstructive pulmonary disease (16%), and hypothyroidism (9%). Chronic kidney disease (CKD) was present in 7% of patients, with 5% having progressed to end-stage renal disease (ESRD). Hyperlipidemia was reported in 7% of the study population. In terms of sex distribution, 57% of patients were male and 43% were female.

These findings are summarized in Table 1.

**Table 1:** Baseline demographic and clinical characteristics of the study population

**Variable**		**Mean ± SD or n (%)**
Age (years)		60.15 ± 9.8
BMI (kg/m^2^)		24.72 ± 5.1
B-Lines POD1 (count)		19.01 ± 7.9
B-Lines POD3 (count)		11.76 ± 7.7
Pre-Op EF (%)		44.70 ± 8.8
Cr (mg/dL)		1.14 ± 0.73
Severely reduced EF (≤ 35%)	Yes	30 (20.3%)
	No	118 (80%)
Prior Heart Failure	Yes	30 (20%)
	No	118 (80%)
Sex	Male	85 (57%)
	Female	63 (43%)
Smoker	Yes	52 (35%)
	No	96 (65%)
HTN	Yes	124 (84%)
	No	24 (16%)
DM	Yes	75 (51%)
	No	73 (49%)
COPD	Yes	24 (16%)
	No	124 (84%)
Hypothyroidism	Yes	13 (9%)
	No	135 (91%)
CKD	Yes	10 (7%)
	No	138 (93%)
HLP	Yes	10 (7%)
	No	138 (93%)
ESRD	Yes	8 (5%)
	No	140 (95%)

Values are presented as mean ± SD or n (%). BMI = body mass index; EF = ejection fraction; CKD = chronic kidney disease; ESRD = end-stage renal disease; COPD = chronic obstructive pulmonary disease; HLP = hyperlipidemia; Cr = creatinine.

### Association between clinical factors and 30-day mortality

Within 1 month after surgery, 22 patients (15%) died. All deaths occurred during the index hospitalization; there were no post-discharge deaths within the 30-day window among patients who were successfully contacted. Univariate logistic regression was used to assess associations between clinical variables and 30-day mortality.

The number of B-lines on POD1 was significantly associated with increased risk of 30-day mortality (OR: 1.091, 95% CI: 1.023–1.162, p = 0.008). Older age showed a trend toward higher mortality (OR: 1.046, 95% CI: 0.997–1.099, p = 0.069), approaching statistical significance.

Male sex was associated with lower odds of death compared to females (OR: 0.433, 95% CI: 0.171–1.094), and this finding was also close to the significance threshold (p = 0.077).

Other variables—including BMI, B-lines on POD3, preoperative EF, creatinine, prior heart failure, and smoking status—did not show statistically significant associations with 30-day mortality. These findings are summarized in Table 2.

**Table 2:** Univariate logistic regression analysis of factors associated with 30-day mortality

**Variable**		**1-Month Mortality**		**OR (95%CI)**	**P-value**
		**Yes**	**No**		
Age (years)		63.77 ± 11.4	59.59 ± 9.3	1.046 (0.997 – 1.099)	0.069
BMI (kg/m^2^)		25.54 ± 5.9	24.45 ± 5	1.041 (0.956—1.133)	0.361
B-Lines POD1 (count)		23.41 ± 6.1	18.47 ± 7.8	1.091 (1.023—1.162)	0.008*
B-Lines POD3 (count)		10.91 ± 6.1	11.88 ± 7.9	0.983 (0.923—1.046)	0.582
Pre-Op EF (%)		41.59 ± 9.9	44.96 ± 8.6	0.959 (0.911—1.009)	0.106
Cr (mg/dL)		1.29 ± 0.792	1.11 ± 0.69	1.349 (0.787—2.314)	0.276
Prior Heart Failure	Yes	7 (32%)	23 (20%)	1.907 (0.697—5.218)	0.209
	No	15 (68%)	94 (80%)		
Sex	Male	9 (41%)	72 (62%)	0.433 (0.171—1.094)	0.077
	Female	13 (59%)	45 (38%)		
Smoker	Yes	6 (27%)	42 (36%)	0.670 (0.244—1.841)	0.437
	No	16 (73%)	75 (64%)		

Values represent odds ratios (OR) with 95% confidence intervals (CI) and corresponding p-values. EF = ejection fraction; Cr = creatinine; HF = heart failure. * indicates p < 0.05.

In the multivariate logistic regression model, which included age, B-lines on POD1, preoperative EF, and sex, the number of B-lines on POD1 remained an independent predictor of 30-day mortality (adjusted OR: 1.090, 95% CI: 1.018–1.167, p = 0.013). This indicates that, even after adjusting for other clinically relevant factors, each additional B-line on POD1 was associated with a 9% increase in the odds of mortality within 30 days.

None of the other variables in the adjusted model reached statistical significance. These findings are summarized in Table 3.

**Table 3:** Multivariate logistic regression analysis of factors associated with 30-day mortality

**Variable**	**Adjusted OR (95% CI)**	**P-value**
Age	1.040 (0.988 – 1.095)	0.137
B-Lines POD1	1.090 (1.018 – 1.167)	0.013*
Pre-Op EF	0.978 (0.926 – 1.032)	0.410
Male Sex	0.521 (0.195 – 1.393)	0.194

Adjusted odds ratios (aOR) with 95% CI are presented from a logistic regression model including age, sex, preoperative EF, and B-lines on POD1. EF = ejection fraction. * Indicates p < 0.05.

### Factors associated with length of hospital stay

The mean length of hospital stay was 11.31 ± 5.14 days, indicating notable variability across the study population. This variation reflects the diverse postoperative recovery trajectories among patients undergoing CABG.

Univariate linear regression analysis showed that higher BMI was significantly associated with longer hospitalization (β = 0.193, p = 0.037), and smokers stayed on average about 2 days longer than non-smokers (β = 2.087, p = 0.029). These findings suggest that both increased body mass index and smoking history may contribute to prolonged postoperative recovery.

Additionally, preoperative EF (β = 0.089, p = 0.102) and history of heart failure (β =  − 2.148, p = 0.070) were not statistically significant but were close to the threshold, suggesting potential clinical relevance that may warrant further investigation.

Other variables, including age and B-lines on POD1 or POD3, were not significantly associated with hospital length of stay. These findings are summarized in Table 4.

**Table 4:** Univariate linear regression analysis of factors associated with length of hospital stay

**Variable**	**B (SE)**	**P-value**
Age	0.007 (0.049)	0.881
BMI	0.193 (0.091)	0.037*
B-Lines POD1	-0.038 (0.059)	0.519
B-Lines POD3	0.000 (0.058)	0.994
Pre-Op EF	0.089 (0.054)	0.102
Cr	-0.762 (0.692)	0.273
Prior Heart Failure	-2.148 (1.176)	0.070
Sex	0.751 (0.949)	0.430
Smoker	2.087 (0.945)	0.029*

Values represent unadjusted β coefficients with standard errors (SE) and p-values. EF = ejection fraction; Cr = creatinine; HF = heart failure. * Indicates p < 0.05.

In the multivariate linear regression model, smoking status (β = 2.20, p = 0.020) and BMI (β = 0.22, p = 0.022) remained independently associated with longer hospital stay. Preoperative EF and B-lines on POD1 were not statistically significant predictors in the adjusted analysis. These findings are summarized in Table 5.

**Table 5:** Multivariate linear regression analysis of factors independently associated with length of hospital stay

**Variable**	**B (SE)**	**P-value**
BMI	0.215 (0.093)	0.022*
B-Lines POD1	0.004 (0.059)	0.953
Pre-Op EF	0.079 (0.053)	0.140
Smoker	2.198 (0.934)	0.020*

Adjusted β coefficients with SE and p-values are presented from a linear regression model including BMI, smoking status, preoperative EF, and B-lines on POD1. EF = ejection fraction. * Indicates p < 0.05.

## Discussion

### Principal findings and comparison with prior studies

In this prospective cohort of post-CABG patients, we found that the extent of pulmonary congestion on lung ultrasound – quantified by B-line counts on POD1 – was significantly associated with short-term mortality. Each additional B-line on POD1 conferred an approximately 9% increase in the odds of 30-day mortality (adjusted OR ≈1.09, p = 0.013). By contrast, B-line counts did not significantly correlate with hospital length of stay in survivors, whereas patient-related factors (notably higher body mass index and active smoking status) were independently associated with prolonged hospitalization. These findings suggest that early postoperative B-lines capture a specific risk phenotype (acute cardiopulmonary decompensation leading to early mortality) distinct from factors that drive protracted recovery. Indeed, our observation that obesity and smoking correlate with longer stays is consistent with prior studies linking elevated BMI and tobacco use to slower postoperative recovery and complications after cardiac surgery [7, 8].

Our results extend the growing evidence that point-of-care lung ultrasound can serve as an early risk stratification tool in surgical patients. Previous research in non-cardiac surgery has shown that immediate postoperative LUS findings in the PACU/ICU correlate with the development of pulmonary complications [9]. Until recently, however, it was unclear whether such ultrasound metrics could prognosticate outcomes in cardiac surgical patients [9]. Fan et al. (2024) addressed this in a cohort of cardiac surgery patients, demonstrating that certain LUS abnormalities – notably pleural effusions > 0.95 cm and multiple consolidations – independently predicted 30-day pulmonary complications [9]. Our study complements and goes beyond those findings by focusing on B-lines as an index of pulmonary edema and showing for the first time that early postoperative B-line burden is linked to increased 30-day mortality after CABG. This aligns with evidence from heart failure and critical care populations: in acute decompensated heart failure, a high B-line count signals residual pulmonary congestion and portends higher mortality [10, 11], and in cardiogenic shock patients, failure to clear B-lines within 24 h is associated with worse 30-day survival [12].

Even in pediatric cardiac surgery, a higher B-line score on POD1 has been strongly associated with extubating failure and prolonged ventilation [5], underscoring that extensive B-lines reflect clinically significant pulmonary edema across patient populations. Taken together, the literature corroborates our principal findings: early postoperative B-lines on lung ultrasound are a meaningful prognostic marker. The lack of association between B-lines and length of stay in our data does not contradict this, but rather suggests that B-lines specifically identify patients at risk of acute, life-threatening complications (e.g. fulminant heart failure or acute respiratory distress) rather than those prone to a slower recovery per se. Patients who survived the initial critical period tended to have hospital stays determined by other factors (such as obesity or smoking-related pulmonary morbidity), whereas B-line counts distinguished those at risk of not surviving the postoperative course.

The observed 30-day mortality rate of 15% warrants contextual interpretation. Shariati Hospital is a large governmental university teaching hospital affiliated with Tehran University of Medical Sciences, serving approximately 35,000 inpatients and 50,000 emergency visits annually. As one of the primary publicly funded referral centers in Tehran, it serves patients with complex, advanced, and often undertreated diseases, many of whom carry substantially higher perioperative risk than typical low-risk elective cohorts reported in Western literature. The elevated mortality rate should therefore be interpreted within this institutional and healthcare context, rather than as a reflection of procedural quality. Importantly, it also provides the outcome variation necessary to evaluate B-lines as a prognostic marker — the primary analytical aim of this study.

The multivariable model warrants further explanation. With only 22 mortality events, the events-per-variable (EPV) ratio imposed a strict limit on model complexity — the widely accepted rule of thumb being approximately one variable per 10 events. Covariate selection was therefore guided by both clinical relevance and univariate screening; all available clinical variables including creatinine, diabetes, COPD, hypertension, and prior heart failure were tested in univariate analysis, and those not included in the final model returned uniformly high p-values. Including additional covariates beyond the EPV threshold would risk overfitting and produce unstable, unreliable estimates. We also acknowledge that several potentially important variables — including bypass duration, number of grafts, postoperative vasoactive requirement, fluid balance, transfusion exposure, and duration of mechanical ventilation — were not collected in our study protocol, which limits the scope of adjustment and represents an important consideration for interpreting our findings.

The final model included four covariates against 22 events, yielding an events-per-variable ratio of approximately 5.5 — which, while below the ideal threshold of 10, reflects a deliberate effort to balance clinical relevance with statistical parsimony given the available data.

### Clinical implications

Our findings have important clinical implications for postoperative management and risk stratification in cardiac anesthesia and intensive care. Lung ultrasound is a non-invasive, bedside technology that provides a real-time window into pulmonary status, offering rapid and accurate detection of pulmonary edema without exposing patients to ionizing radiation [5]. Integrating a simple 8-zone lung ultrasound exam into routine postoperative care could enable anesthesiologists and intensivists to identify high-risk patients early. A patient with an abnormally high B-line count on POD1 after CABG should prompt heightened vigilance and possibly proactive intervention. For instance, marked B-line proliferation (signifying diffuse extravascular lung water) might warrant prompt optimization of fluid management (e.g. diuresis or fluid restriction) and a thorough evaluation for cardiac dysfunction or incipient heart failure. It is noteworthy that traditional monitoring can underestimate subclinical pulmonary congestion; one study in acute heart failure found that adding LUS B-line assessment provided significant prognostic information beyond clinical signs like crackles or peripheral edema [10]. Thus, incorporating lung ultrasound may improve risk stratification compared to relying on physical exam and chest X-ray alone.

In practical terms, lung ultrasound-based risk assessment could complement existing postoperative scoring systems. Patients flagged by a high B-line count could be considered for closer hemodynamic monitoring, delayed step-down from ICU, or early therapeutic measures to mitigate pulmonary congestion. This approach resonates with prior recommendations in the literature [9]. By identifying occult pulmonary edema early, clinicians have an opportunity to intervene before full-blown respiratory failure or other complications ensue.

Furthermore, our study reinforces the importance of addressing baseline risk factors: the association of smoking and obesity with prolonged stays highlight the need for preoperative optimization (smoking cessation, weight management) to improve recovery [7]. Nevertheless, those measures alone do not guard against acute postoperative decompensation – a gap which bedside lung ultrasound may help fill.

In summary, the use of B-lines on LUS as an early warning marker is a novel, clinically actionable concept: it equips providers with timely information on a patient’s cardiopulmonary status, enabling a tailored escalation of care for those in jeopardy, while potentially sparing low-congestion patients from unnecessary interventions. With growing availability of handheld ultrasound devices, this strategy is both feasible and readily implementable in the cardiac ICU setting, offering a practical enhancement to postoperative risk stratification and management.

### Limitations and future directions

This study has several limitations. First, it was conducted at a single center with a moderate sample size (148 patients) and correspondingly few mortality events, which may limit the statistical power and generalizability of the findings. Second, our analysis focused on elective CABG patients; the results may not directly extrapolate to emergent surgeries or other cardiac procedures. Third, we did not systematically evaluate other sonographic abnormalities (such as pleural effusions or consolidations) in our cohort. These lesions were shown in prior studies to predict pulmonary complications [9] and might provide complementary prognostic information beyond B-lines alone.

Furthermore, while we included 30-day mortality as a primary outcome, we did not track the exact time of death. This limited our ability to perform time-to-event analyses such as Cox regression or Kaplan–Meier survival curves. Similarly, heart rate and hemodynamic trends were not systematically recorded, which may have provided complementary physiological context to the B-line findings.

Furthermore, intraoperative parameters such as bypass time, number of grafts, and vasoactive requirements were not systematically captured in our protocol; future studies should incorporate comprehensive perioperative procedural data from the outset. Furthermore, preoperative cardiogenic shock status and use of mechanical circulatory support were not systematically collected and may represent unmeasured confounders.

The exact timing of lung ultrasound examinations relative to extubation was not systematically recorded, which may have introduced variability in the B-line measurements across patients.

The multivariable model was limited to four covariates due to EPV constraints, and several intraoperative and postoperative variables were not collected in our protocol; the rationale for model selection is discussed in detail above. Penalized regression methods and internal validation techniques such as bootstrapping were not employed; future larger studies should incorporate these more robust modeling strategies.

Furthermore, the per-unit linear interpretation of the B-line odds ratio assumes a linear dose–response relationship across the entire range of B-line counts. This assumption has not been formally tested, and threshold behavior — where mortality risk accelerates beyond a certain B-line burden — remains a plausible alternative. Exploring nonlinear relationships or categorical groupings was not feasible given the small number of outcome events; future larger studies should specifically investigate clinically meaningful B-line cutoff values.

Nine patients (5.7% of the originally enrolled cohort of 157) were lost to follow-up and excluded from analysis prior to any modeling. Formal best-case and worst-case sensitivity analyses assuming different outcome scenarios for these patients were not performed. However, given that they represent a small proportion of the cohort and that the primary finding demonstrated a statistically significant association with a clear effect size, it is unlikely that any reasonable assumption regarding their outcomes would materially alter the direction or significance of the results.

The LOS analysis used linear regression without formal verification of the normality assumption. Alternative approaches such as log-transformation or negative binomial regression may be more robust for skewed LOS distributions and are recommended for future studies. However, as LOS was a secondary outcome and B-lines were not significantly associated with it regardless of modeling approach, this limitation does not affect the primary conclusions of the study.

Intra-observer variability in B-line quantification was not formally assessed, as repeated measurements on the same patient at the same time point were not performed. This represents a limitation of the reproducibility assessment in our study.

Despite multivariable adjustment, the possibility of residual confounding by unmeasured variables, including disease severity, medication use, socioeconomic factors, and perioperative parameters not captured in our protocol — cannot be excluded and should be considered when interpreting the findings.

Another consideration is that postoperative fluid management was not controlled in our observational design; variations in diuretic use or fluid administration could confound the relationship between B-line burden and outcomes. Finally, we only examined short-term endpoints (30-day mortality and in-hospital metrics). The prognostic value of early B-lines for longer-term outcomes (such as one-year survival or quality of recovery) remains unknown.

Despite these limitations, our findings open several avenues for future research. Larger multi-center studies should be undertaken to validate the association between early postoperative B-lines and mortality, and to determine whether a specific B-line threshold (or change in B-line count over time) yields the highest predictive accuracy. It would also be valuable to explore integration of lung ultrasound into formal risk models for postoperative outcomes – for example, adding LUS metrics to existing cardiac surgery risk indices to see if prediction of complications or death is improved. An important next step will be interventional studies to test whether managing patients based on lung ultrasound findings can actually improve outcomes. A randomized trial could investigate protocols where high-B-line patients receive aggressive decongestive therapy or extended ICU monitoring versus standard care. Such research would clarify if the LUS marker is not only predictive but also modifiable in its impact.

Monitoring daily LUS in the postoperative period could similarly distinguish patients who are recovering versus those who are failing to resolve pulmonary edema. Future studies should examine whether changes in B-lines from POD1 to POD3 (the latter time point was measured in our study but not significantly predictive) carry meaning – for instance, persistent or worsening congestion might identify patients who need rescue therapies.

Finally, exploration of lung ultrasound in broader contexts (such as valve surgery or heart failure patients managed in the surgical ICU) will help determine the generalizability of using B-lines as a postoperative risk stratification tool. By addressing these questions, the field can better define the role of lung ultrasound in guiding perioperative care and potentially translate this readily accessible imaging modality into improved patient outcomes after cardiac surgery.

## Conclusion

In this prospective cohort of patients undergoing coronary artery bypass grafting, early postoperative pulmonary congestion—quantified by B-line burden on POD1 lung ultrasound—was an independent predictor of 30-day mortality. Each additional B-line on POD1 increased the risk of death, underscoring the prognostic value of early lung ultrasound as a non-invasive bedside tool for risk stratification. In contrast, B-line counts on POD3 were not significantly associated with mortality, suggesting that early rather than delayed congestion markers carry prognostic weight. Moreover, B-lines were not related to hospital length of stay, which was instead influenced by baseline patient factors such as smoking and elevated body mass index.

These findings support the use of routine lung ultrasound on the first postoperative day to identify high-risk patients and guide timely optimization of fluid and cardiopulmonary management. Larger multicenter studies are needed to confirm these results, define clinically meaningful thresholds, and determine whether targeted interventions based on early ultrasound findings can improve postoperative outcomes after cardiac surgery.

## Data Availability

The datasets collected and analyzed during the present study are maintained in a database and can be obtained from the corresponding author upon reasonable request.

## Abbreviations

BMI: Body mass index
CABG: Coronary artery bypass grafting
CI: Confidence interval
CKD: Chronic kidney disease
COPD: Chronic obstructive pulmonary disease
Cr: Creatinine
EF: Ejection fraction
ESRD: End-stage renal disease
HF: Heart failure
HLP: Hyperlipidemia
HTN: Hypertension
ICU: Intensive care unit
LUS: Lung ultrasound
OR: Odds ratio
PACU: Post-anesthesia care unit
PAP: Pulmonary artery pressure
SD: Standard deviation
SPSS: Statistical package for the social sciences

## References

[1] Gargani L. Lung ultrasound: a new tool for the cardiologist. Cardiovasc Ultrasound. 2011;9(1):6. 10.1186/1476-7120-9-6 PMC3059291 21352576

[2] Anile A, Russo J, Castiglione G, Volpicelli G. A simplified lung ultrasound approach to detect increased extravascular lung water in critically ill patients. Crit Ultrasound J. 2017;9(1):13. 10.1186/s13089-017-0068-x PMC5469722 28612302

[3] Sakamoto T, Asanuma T, Sasaki H, Kawahara H, Uchida K, Endo A, et al. Diagnostic value of lung ultrasound B-lines for evaluating left ventricular filling pressure. Cardiovasc Ultrasound. 2025;23(1):6. 10.1186/s12947-025-00341-7 PMC11995549 40223118

[4] Imanishi J, Maeda T, Ujiro S, Masuda M, Kusakabe Y, Takemoto M, et al. Association between B-lines on lung ultrasound, invasive haemodynamics, and prognosis in acute heart failure patients. Eur Heart J Acute Cardiovasc Care. 2023;12(2):115–23. 10.1093/ehjacc/zuac158 PMC9910565 36548965

[5] Rezende RQ, Lopes CLS, Ricachinevsky CP, Piva JP. Lung ultrasound accuracy to predict ventilatory outcomes after paediatric cardiac surgery. Cardiol Young. 2025;35(5):1011–5. 10.1017/S1047951125001775 40384370

[6] Charoenitthikun N, Tuchinda L, Somcharoen P. Correlation between pulmonary artery pressure and lung ultrasound score in postoperative cardiac surgical patients. Thai J Anesthesiol. 2016;42(4):235–42.

[7] Ji Q, Zhao H, Mei Y, Shi Y, Ma R, Ding W. Impact of smoking on early clinical outcomes in patients undergoing coronary artery bypass grafting surgery. J Cardiothorac Surg. 2015;10(1):16. 10.1186/s13019-015-0216-y PMC4322818 25654995

[8] Saker Z, Saab M, Rabah A. The impact of body mass index on the duration of hospital stay after cardiac surgery. Cureus. 2025;17(5):e84985. 10.7759/cureus.84985 PMC12204642 40585631

[9] Fan G, Zhang F, Shan T, Jiang Y, Zheng M, Zang B, et al. Association of point-of-care lung ultrasound findings with 30-day pulmonary complications after cardiac surgery: a prospective cohort study. Heliyon. 2024;10(10):e31293. 10.1016/j.heliyon.2024.e31293 PMC11133817 38813155

[10] Araiza-Garaygordobil D, Gopar-Nieto R, Martínez-Amezcua P, Cabello-López A, Manzur-Sandoval D, García-Cruz E, et al. Point-of-care lung ultrasound predicts in-hospital mortality in acute heart failure. QJM. 2021;114(2):111–6. 10.1093/qjmed/hcaa298 33151302

[11] Suhardi KF, Prasetyo A, Malik R, Pauliana, Viazelda AT. Quantification of residual pulmonary congestion defined by B-line findings on lung ultrasound to predict cardiovascular events in acute heart failure: a systematic review and meta analysis. Indian Heart J. 2025;77(4):258–66. 10.1016/j.ihj.2025.05.003 PMC12327527 40334938

[12] Tavazzi G, Colombo CNJ, Pagnesi M, Bertaina M, Montisci A, Frea S, et al. Lung ultrasound and mortality in a cardiogenic shock population: a prospective registry-based analysis. Eur J Heart Fail. 2025. 10.1002/ejhf.3692. PMC12765037 40444572

